# Serial analysis of *ESR1* mutations in cell-free DNA from hormone receptor-positive, HER2-negative metastatic breast cancer during palliative endocrine therapy

**DOI:** 10.3389/fonc.2025.1709317

**Published:** 2026-01-06

**Authors:** Soo Yeon Baek, Meehyang Hong, Seungah Lee, Seulgi Joo, JungPil Jang, Chinkoo Jung, Hyein Lee, Tae-Kyung Yoo, Il Yong Chung, Hee Jeong Kim, Beom Seok Ko, Jong Won Lee, Byung Ho Son, Hyehyun Jeong, Jae Ho Jeong, Jin-Hee Ahn, Kyung Hae Jung, Sung-Bae Kim, Hee Jin Lee, Gyungyub Gong, Sae Byul Lee, Jisun Kim

**Affiliations:** 1 Division of Breast Surgery, Department of Surgery, University of Ulsan College of Medicine, Asan Medical Center, Seoul, Republic of Korea; 2 Department of Surgery, Ajou University School of Medicine, Suwon, Republic of Korea; 3 Genopeaks Co., Ltd., Seoul, Republic of Korea; 4 Asan Institute for Life sciences, Seoul, Republic of Korea; 5 Department of Oncology, Asan Medical Center, University of Ulsan College of Medicine, Seoul, Republic of Korea; 6 Department of Pathology, Asan Medical Center, University of Ulsan College of Medicine, Seoul, Republic of Korea

**Keywords:** breast cancer, cell-free DNA, circulating tumor DNA, endocrine therapy, ESR1 mutation

## Abstract

**Background:**

Activating mutations in *ESR1* gene are a known mechanism of endocrine resistance. We analyzed *ESR1* mutations in cell-free DNA (cfDNA) from serially collected blood from hormone receptor-positive, human epidermal growth factor receptor 2-negative metastatic breast cancer patients to investigate their impact on outcomes.

**Methods:**

A total of 268 cfDNA samples serially collected from 25 patients who underwent palliative endocrine therapy were examined. Seven *ESR1* hotspot mutations were tested in cfDNA using droplet digital polymerase chain reaction. Progression-free survival (PFS) was analyzed using the Kaplan–Meier method. Blood *ESR1* mutation (b*ESR1*) results were correlated with clinical response.

**Results:**

Among 25 patients, baseline b*ESR1* mutation was negative for all patients (0/4) and 64.0% (16/25) was b*ESR1*-positive at any time. b*ESR1* positivity did not affect PFS. Those whose b*ESR1* cleared in subsequent cfDNA exhibited longer PFS. Among patients with clinical progression, 57.1% (8/14) were b*ESR1*-positive, and four had b*ESR1* detected before clinical progression.

**Conclusions:**

We observed worse outcomes in patients with persistent b*ESR1* detection during first-line endocrine therapy and then sustained positive. b*ESR1* was detected prior to clinical progression in half of patients. Our study suggests the benefit of b*ESR1* monitoring during palliative endocrine therapy.

## 1 Introduction

Approximately 80% of the breast cancers (BCs) express estrogen receptor (ER) (1). Endocrine therapy (ETx) significantly reduces the recurrence and mortality rates of ER-positive BC (2). However, even after receiving ETx, patients with ER-positive BC have a persistent risk of recurrence for up to 20 years after diagnosis (3). Notably, endocrine resistance remains a major clinical challenge in treating ER-positive BC. Mutations in ligand binding site in estrogen receptor 1 (*ESR1*) gene are known mechanism of endocrine resistance in hormone receptor-positive BC, particularly under aromatase inhibitor (AI) therapy. The presence of these mutations is associated with poor prognosis (4, 5).

*ESR1* mutation confer endocrine resistance by altering the structure and function in the ligand binding domain of ERα (6). *ESR1* mutations are rare in primary BC but are enriched in metastatic BC, particularly in patients previously exposed to AIs (7, 8). Several studies have revealed the role of *ESR1* mutations as prognostic biomarkers as well as negative predictive factor of AIs (4, 5). A study that conducted circulating tumor DNA (ctDNA) analysis of plasma samples from patients in the “Study of Faslodex versus Exemestane with or without Arimidex (SoFEA)” trial showed that patients with *ESR1* mutations had improved progression-free survival (PFS) when treated with fulvestrant compared to those treated with exemestane (9). Fulvestrant is a first-in-class selective estrogen receptor degraders (SERD). However, fulvestrant has a low bioavailability and is delivered via intramuscular injections. In addition, a preclinical study reported reduced efficacy of fulvestrant due to a specific type of *ESR1* mutation (10). To overcome this limitation, orally bioavailable SERDs have been developed. In the EMERALD trial (NCT03778931), elacestrant first demonstrated a significant improvement in PFS in patients with *ESR1* mutations (11). Imlunestrant (EMBER-3 trial, NCT04975308) also showed significantly longer PFS compared with standard therapy in patients harboring *ESR1* mutations (12). Recently, in SERENA-6 trial (NCT04964934), switching to camizestrant after the detection of *ESR1* mutations resulted in significantly longer PFS than continuing AI therapy (13). This finding implies the importance of serial monitoring of *ESR1*mutation throughout the ETx beyond one-time testing *ESR1*. As this population of ER-positive HER2-negative breast cancer exhibit indolent biologic nature, ctDNA monitoring may provide window of opportunity period longer enough to actually increase treatment outcome compared to following radiologic response only.

ctDNA analysis from cell-free DNA (cfDNA) has emerged as an important tool for detecting these clinically relevant mutations. In some patients with metastases, it may not be possible to obtain metastatic tissues. Liquid biopsy using ctDNA from cfDNA is a non-invasive method for detecting mutations. *ESR1* mutations are mostly acquired under therapeutic pressure during ETx, and exists in subclones. ctDNA testing detects approximately 20–40% of *ESR1* mutations in metastatic BC (7–9) and is recommended as a primary test for *ESR1* mutation detection (14). However, evidence for the serial monitoring of ctDNA during treatment is limited. In a clinical trial evaluating the efficacy of inhibitors of cyclin-dependent kinases 4 and 6 (CDK4/6i) in ER-positive BC, short-term reduction in *PIK3CA* ctDNA levels predicted prognosis and treatment response (15). It has also been reported that ctDNA increases during treatment identified disease progression in a significant proportion of patients before detection in radiological studies using longitudinal ctDNA analysis (16). In a randomized phase III PADA-1 trial (NCT03079011), patients with ER-positive/human epidermal growth factor receptor 2 (HER2)-negative advanced BC receiving first-line AI and CDK4/6i therapy were recruited and monitored for rising blood *ESR1* (b*ESR1*) mutations (17). Switching ETx after the elevation of the b*ESR1* mutations and before clinical tumor progression significantly improved PFS in these patients. While waiting for overall survival analysis, these results suggest the clinical benefit of ctDNA monitoring on top of current standard radiological response monitoring in selected group of patients (18).

In this study, we analyzed b*ESR1* mutations in serial cfDNA of patients with hormone receptor -positive/HER2-negative metastatic BC collected at multiple time points corresponding to radiological imaging. We aimed to track b*ESR1* mutation changes during palliative treatment and compare mutation detection and the time of clinical disease progression. We also analyzed the association between b*ESR1* mutations and PFS.

## 2 Methods

### 2.1 Patients

**Figure 1:**
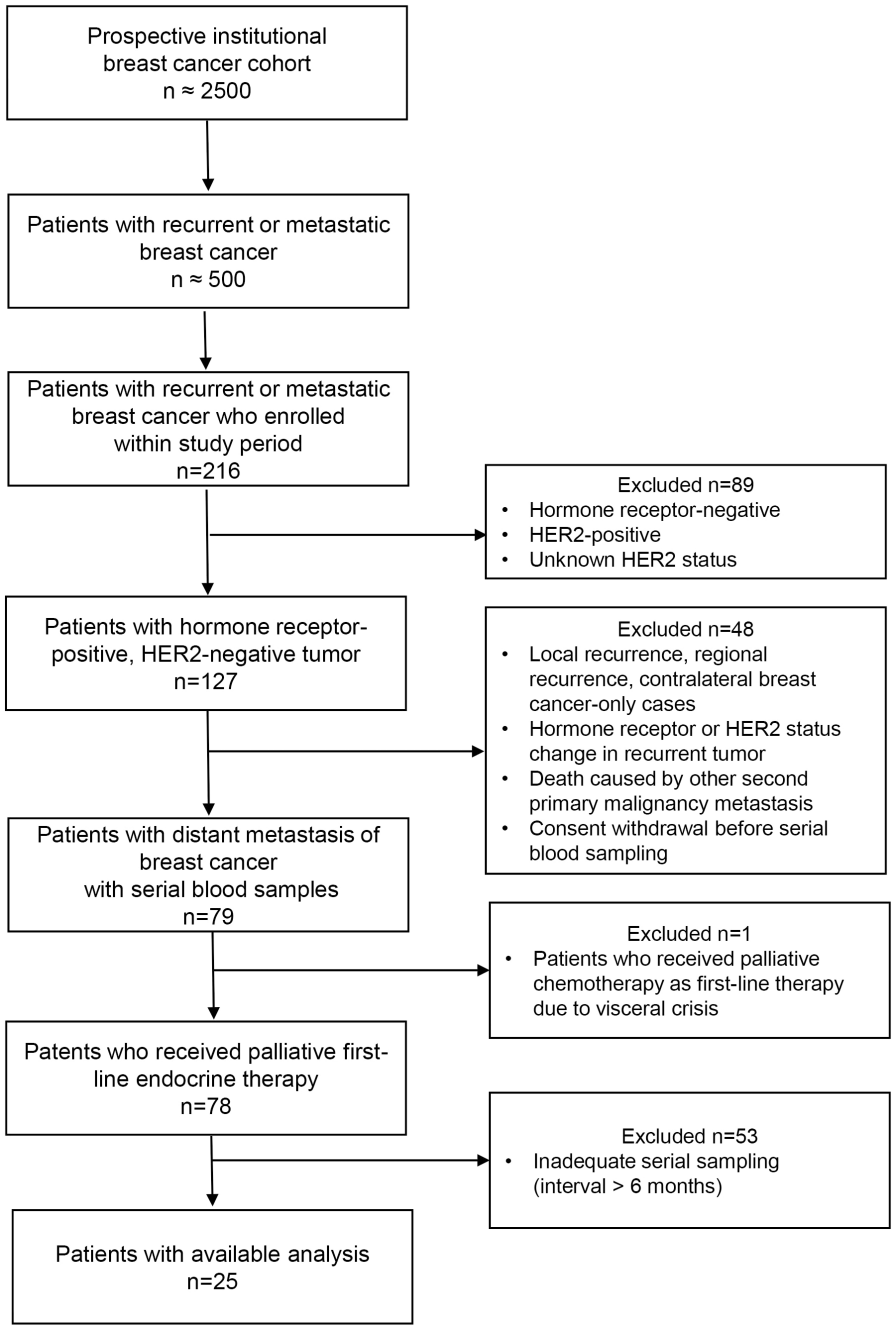
Flow diagram of patient selection criteria.

The overall cohort consists of patients with breast cancer who have been enrolled consecutively since 2016, either at the time of initial diagnosis or at the time of diagnosis of recurrent or metastatic disease during treatment at Asan Medical Center, Seoul, Republic of Korea. The cohort currently includes more than 2,500 patients, of whom approximately 500 have metastatic breast cancer, and has been maintained to date. For the present study, we included patients with metastatic breast cancer who had hormone receptor-positive and HER2-negative disease and had been treated with palliative ETx. Among them, only those with every 2–6months of serially collected blood samples available were included in the experimental analyses. Patient enrollment occurred from August, 2017 to March, 2023. Two hundred sixteen patients with recurrent or metastatic BC were identified from the cohort. All patients underwent prior adjuvant ETx and were considered eligible if primary tumor tissue was available, along with plasma samples at time of metastasis, and every 2–6months thereafter during palliative ETx. Twenty-five were included in this study (**Figure 1**). We performed a retrospective analysis of cases selected from a prospectively collected cohort. Clinicopathologic data and radiologic images were retrospectively reviewed using electronic medical records.This study was approved by the Institutional Review Board of the Asan Medical Center (IRB No. 2019-1480) and written informed consent had been prospectively obtained. HR positivity was defined as nuclear staining ≥1% or an Allred score of 3–8 based on the results of immunohistochemistry (IHC) staining for the ER or progesterone receptor. HER2 positivity was defined as 3+ by IHC staining, HER2 gene amplification by fluorescence *in situ* hybridization (FISH), or silver-enhanced *in situ* hybridization (SISH). HER2 grade 2+ (equivocal) without FISH or SISH testing was defined as an unknown HER2 status and was excluded. Primary endocrine resistance was defined as relapse during the first two years of adjuvant ETx or progression during the first six months of first-line ETx. Secondary/acquired resistance was defined as disease relapse while on adjuvant ETx but after the first two years, relapse within 12 months of completing adjuvant ETx, or progressive disease ≥6 months after initiating first-line ETx (19). Radiological imaging and clinical evaluations were performed approximately every 2–4 months in accordance with image and disease assessment guidelines for metastatic breast cancer (19). The clinical response to palliative therapy was assessed according to the physician’s assessment from the radiological evidence of changes in the gross disease burden. We analyzed mutations in plasma samples both at time of the radiological assessment, and also in between the radiologic imagings.

### 2.2 Sample collection and processing

Blood collection was planned at intervals of 2–6 months, according to each patient’s hospital visit and blood sampling schedule. All patients’ blood sampling and plasma preparation followed the standardized protocol of the ‘clinical research center laboratory’. Blood samples were collected in EDTA blood collection tubes. The samples were processed within one hour of collection. Plasma was obtained by double centrifugation at 1600 ×g for 10 minutes at 4°C and 3000 ×g for 10 minutes at 4°C. The plasma was stored at -80°C prior to cfDNA extraction.

### 2.3 Cell-free DNA extraction

cfDNA was extracted from 2 mL of processed plasma. The extraction was performed using the QIAamp Circulating Nucleic Acid Kit (Qiagen, Hilden, Germany) according to the manufacturer’s protocol.

### 2.4 Droplet digital PCR

*ESR1* mutations were defined as seven hotspot mutations (D538G, Y537C, Y537S, Y537N, L536R, S463P, and E380Q) among missense mutations in the ligand-binding domain (Codon 310-547). *ESR1* hotspot mutations in the ligand-binding domain were detected in cfDNA by droplet digital PCR (ddPCR) using an *ESR1* mutations detection kit (Genopeaks Co., Ltd., Seoul, Republic of Korea). We performed ddPCR on cfDNA extracted from plasma for *ESR1* mutation detection. To determine the limit of detection of ddPCR, the following method was established: *ESR1* mutant plasmid DNA samples were serially diluted to concentrations of 0.5%, 0.3%, 0.1%, 0.05%, and 0.01% with *ESR1* wild-type plasmid DNA, and each concentration was subjected to three replicates of ddPCR testing. We confirmed that up to 0.01% (three copies) of mutant plasmid DNA could be detected. Typically, the input DNA amount was 30 ng; however, in cases where the results were ambiguous, the input DNA amount was increased threefold for a re-test to ensure accurate results. Due to insufficient sample from one patient, three ddPCR results that required re-examination could not be performed and were excluded from this analysis. We re-examined samples that were clinically ambiguous or inconsistent with the patient’s course.

### 2.5 Statistical analysis

PFS was defined as the interval from the start of palliative first-line ETx to the time of disease progression or death from any cause. PFS analyses were performed using the Kaplan–Meier method and compared using the log-rank test. Hazard ratios (HRs) and 95% confidence intervals (CIs) for PFS were estimated using univariable Cox proportional hazards regression. The proportional hazards assumption was visually evaluated using log-survival plots. No major violations were detected. We generated swimmer plots to illustrate the clinical course of the patient and *ESR1* mutation detection. Means were compared using an independent *t*-test for normally distributed continuous variables. Clearance of *ESR1* mutation was defined as a mutation detected at a specific time point but not detected in subsequent samples. Exploratory multivariable Cox proportional hazards models including b*ESR1* status and key clinical factors (visceral metastasis or CDK4/6i use) were also fitted to assess their association with PFS. All reported P values were two-sided, and *p* < 0.05 was considered statistically significant. All statistical analyses were performed using the IBM SPSS Statistics for Windows, version 25.0 (IBM Corp., Armonk, NY, USA) and R, version 4.3.2 (The R Project for Statistical Computing, Vienna, Austria). All laboratory assays were conducted at Genopeaks Co., Ltd. in a blind manner, and the data analysis and interpretation of the results were performed independently by the academic investigators to ensure objectivity.

## 3 Results

### 3.1 Characteristics of the patients and analyzed blood samples

Among the 216 prospectively enrolled metastatic BC patients within the cohort, 25 patients met the predefined requirement of serial blood sampling every 2–6 months during first-line ETx and were included in the analysis (**Figure 1**). Their clinical outcomes were analyzed retrospectively based on medical record review. Serial blood samples were collected according to each patient’s hospital visit schedules. A total of 268 samples were analyzed with the mean number of samples 10.7 ± 3.1 per patient. The mean interval of blood sampling was 3.3 ± 2.9 months, with most samples collected every 2–4 months. Only one patient had an extended sampling interval of 6 months, according to the physician’s follow-up schedule. cfDNA was extracted for the analyses from the processed/storage plasma as described above. Patient characteristics are summarized in **Table 1**. The mean age at the initial diagnosis was 45.2 years. Five (20.0%) patients had *de novo* distant metastases. Adjuvant ETx was administered in patients in stage I–III or those in stage IV who had their metastatic lesions disappear after neoadjuvant chemotherapy and subsequently underwent curative surgery. Among these patients, 13 (61.9%) received tamoxifen and 5 (23.8%) received tamoxifen with ovarian suppression. There were 2 (9.5%) who received AI prior to palliative ETx. Most patients (90.9%) exhibited secondary endocrine resistance. At the time of distant metastasis, 16 patients (64.0%) were premenopausal. Most premenopausal patients underwent bilateral salpingo-oophorectomy before starting palliative ETx; however, one premenopausal patient underwent bilateral salpingo-oophorectomy four months after starting palliative ETx with ovarian suppression. Eleven patients had bone-only metastasis and 32% (8/25) had visceral metastases.

**Table 1:** Patients characteristics (n = 25).

Characteristic	n (%)
Age at diagnosis (years; mean ± SD)	45.2 ± 7.8
Menopausal status at distant metastasis	
Premenopause	16 (64.0)
Postmenopause	9 (36.0)
Distant metastasis	
Recurrent	20 (80.0)
* De novo*	5 (20.0)
Previous treatment	
Adjuvant endocrine therapy	
Tamoxifen^a^	13 (61.9)
Tamoxifen + ovarian suppression	5 (23.8)
Aromatase inhibitor	2 (9.5)
Tamoxifen → Aromatase inhibitor extension	1 (4.8)
Chemotherapy	
Neoadjuvant	8 (40.0)
AC 4 cycles → Taxane 4 cycles	7
FEC 3 cycles → Taxane 3 cycles^b^	1
Adjuvant	5 (25.0)
AC 4 cycles	3
AC 4 cycles → Taxane 4 cycles	1
CAF 6 cycles	1
Neoadjuvant and adjuvant	1 (5.0)
Neoadjuvant AC 4 cycles → adjuvant Taxane 4 cycles	
No	6 (30.0)
Adjuvant radiotherapy	
Yes	15 (75.0)
No	5 (25.0)
Endocrine resistance status	
Primary endocrine resistance	2 (9.1)
Secondary endocrine resistance	20 (90.9)
Sensitivity (*de novo* metastasis)	3
Number of metastatic sites	
1	18 (72.0)
2	5 (20.0)
≥ 3	2 (8.0)
Site of distant metastasis	
Non-visceral metastasis	12 (48.0)
Bone only	11
Lymph nodes	1
Visceral metastasis	8 (32.0)
Liver	3
Lung	2
Liver and lung	1
Pleura	2
Non-visceral & visceral metastases	5 (20.0)
Palliative first-line endocrine therapy regimen	
CDK4/6i^c^ + Aromatase inhibitor^d^	18 (72.0)
CDK4/6i^c^ + Fulvestrant	1 (4.0)
Aromatase inhibitor ± ovarian suppression^e^	4 (16.0)
Fulvestrant	2 (8.0)
Palliative surgery	
No	22 (88.0)
Yes	3 (12.0)
Palliative radiotherapy	
No	16 (64.0)
Yes	9 (36.0)

Data are shown as number (%), not otherwise specified.

SD, standard deviation; AC, anthracycline and cyclophosphamide; FEC, fluorouracil, epirubicin, and cyclophosphamide; CAF, cyclophosphamide, adriamycin and fluorouracil; CDK4/6i, inhibitor of cyclin-dependent kinases 4 and 6.

a One patient with *de novo* metastasis in the lymph node underwent surgery with curative intent after neoadjuvant chemotherapy, and tamoxifen was used as adjuvant ETx.

b Neoshorter study (NCT02001506).

c Palbociclib was used as a CDK4/6 inhibitor in all cases.

d In combination with CDK4/6i, letrozole was used as an aromatase inhibitor in all cases.

e One premenopausal patient initially began treatment with an aromatase inhibitor and gonadotropin-releasing hormone agonist. After 4 months of treatment, the patient underwent bilateral salpingo-oophorectomy and then used only aromatase inhibitor.

**Figure 2:**
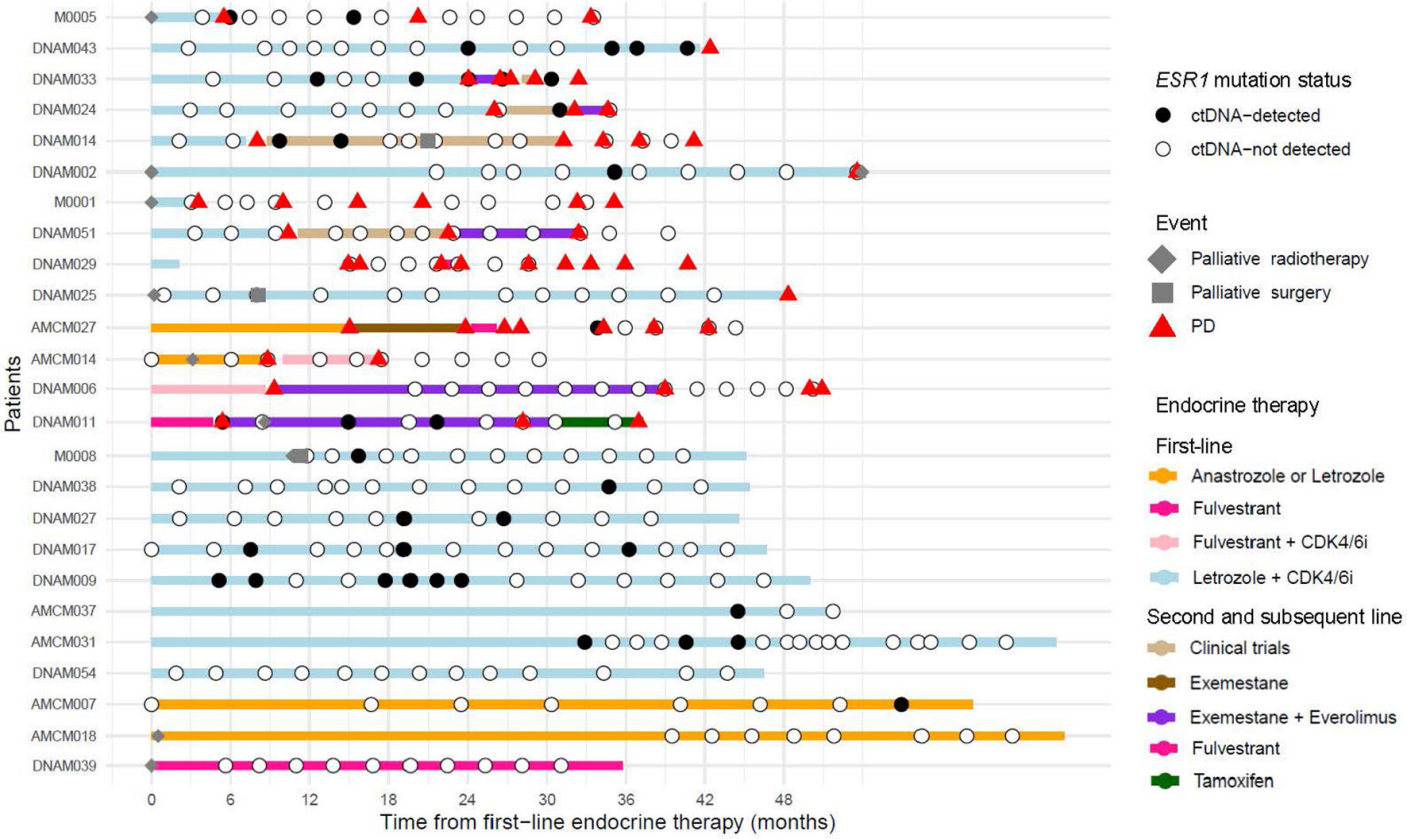
Swimmer plot presenting *ESR1* mutation status and clinical information of individual patients after palliative first-line endocrine therapy. ctDNA circulating tumor DNA, PD progressive disease, CDK4/6i inhibitors of cyclin-dependent kinases 4 and 6.

Most patients (72.0%) received CDK4/6i + AI as palliative first-line ETx, followed by AI (16.0%), fulvestrant (8.0%), and CDK4/6i + fulvestrant (4.0%) (**Table 1**). The ETx regimen for each patient is shown in **Figure 2**. Four patients were enrolled in the oral SERD clinical trials for the further lines of ETx. Palliative surgery and radiotherapy were performed in three (12.0%) and nine (36.0%) patients, respectively. Two patients underwent both palliative radiotherapy and surgery. Two patients underwent palliative breast cancer surgery. Although palliative surgery is not part of the standard treatment at our institution, these cases represented unique circumstances in which the patients demonstrated a radiologic complete response after prolonged palliative systemic therapy. Following multidisciplinary team discussions, local treatment was performed for the residual breast lesion, which was the only remaining site of disease. Another patient underwent lung surgery for pathologic confirmation of pulmonary lesions aiming for diagnostic purposes rather than palliative surgery. The overall median PFS was 43.4 months (range, 3.6–69.3 months).

### 3.2 *bESR1* mutation during palliative endocrine therapy

We performed longitudinal ctDNA analyses for each patient. The median follow-up period from first-line ETx initiation was 44.6 months (range, 32.5–69.3 months). Baseline cfDNA at the initiation of palliative ETx was analyzed in four patients, and all were negative for b*ESR1* mutations. Among them, three patients had *de novo* metastasis. The remaining patient had received adjuvant tamoxifen plus ovarian suppression. The disease-free interval for this patient was 31.2 months. Among the 25 patients, b*ESR1* mutations were detected in 16 (64.0%) at any time point during first-line ETx. Among them, there were 3 (18.8%) patients whose *ESR1* mutations were continuously detected until the last cfDNA examination. Among 268 samples analyzed, there were 53 samples in which *ESR1* mutations were detected. The most common mutation was D538G (17/53), followed by S643P (14/53), Y537C (12/53), Y537N (6/53), Y537S (2/53), and E380Q (2/53). A summary of mutation-specific characteristics, including copy number ranges and associated temporal patterns, is provided in **Supplementary Table S1**. Multiple mutations were detected in 11 (68.8%) patients.

### 3.3 *bESR1* mutation and clinical courses

**Figure 2** provides the longitudinal clinical courses and treatment timelines of all 25 patients, including the timing of b*ESR1* mutation detection. Among the patients that were b*ESR1*-positive, eight (50%) showed clinical progression, of which 4 cases had b*ESR1* detected prior to clinical progression (DNAM043, DNAM033, DNAM002, and AMCM027). The mean lead time was 7.6 months. In the remaining 4 patients (M005, DNAM024, DNAM014, and DNAM011), mutations were detected simultaneously or after clinical progression. Remarkably, in all patients responding to AI therapy, the b*ESR1* mutation was never detected, or if initially detected, subsequently converted negative. There were three patients with stable disease in whom the b*ESR1* mutation was never detected.

**Supplementary Figure S1** depicts clinical courses with corresponding b*ESR1* status after ETx initiation. A total of 19 patients received adjuvant ETx with a tamoxifen. Most of these patients (18/19) subsequently underwent AI-based palliative treatment. Of the 18 patients who subsequently received AI-based palliative therapy, disease progression was observed in 11 patients. b*ESR1* mutations were detected in 7 of the patients in whom the disease progressed. In these patients, b*ESR1* mutation was first detected at 6.0 to 35.1 months after the commencement of palliative AI therapy. Of the two patients who received adjuvant ETx with AI, one (DNAM011) exhibited a b*ESR1* mutation identified 48 months after adjuvant treatment initiation. This patient maintained stable disease for 4 months on palliative fulvestrant before experiencing disease progression. In both patients with primary endocrine resistance (DNAM051 and DNAM039), no b*ESR1* mutation was found. Among the 20 patients with secondary endocrine resistance, b*ESR1* mutations were detected in 70%. Furthermore, b*ESR1* mutation was also detected in 2 out of 3 patients with endocrine-sensitive *de novo* metastasis (M0008 and AMCM007; **Figure 2**), of whom the patient who had subsequent samples (M0008) turned bE*SR1* negative and had stable disease for more than 36 months.

### 3.4 Survival analysis

**Figure 3:**
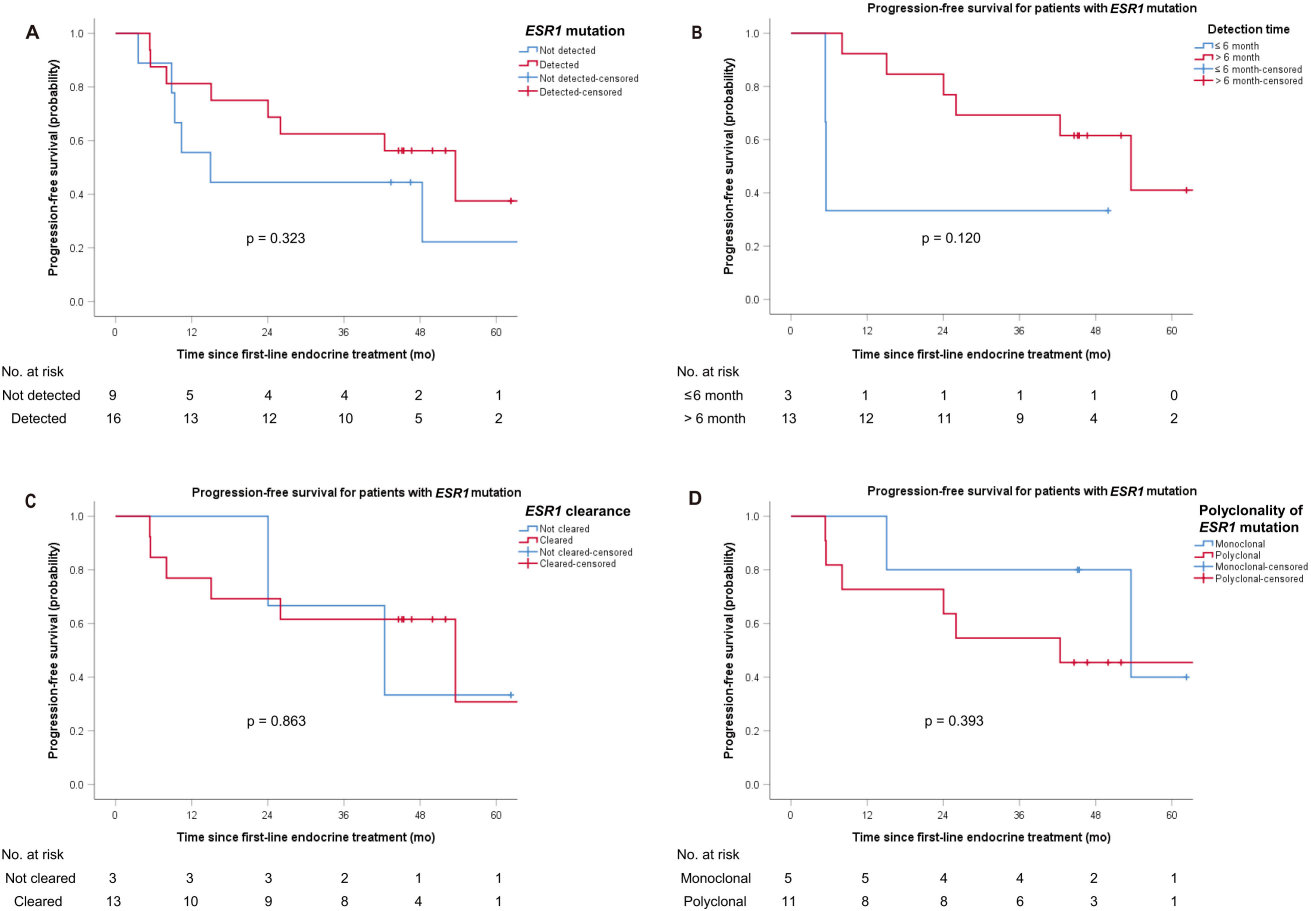
Kaplan–Meier curves of progression-free survival. **(A)** according to *ESR1* mutation status. **(B)** according to *ESR1* mutation detection time. **(C)** according to *ESR1* clearance. **(D)** according to polyclonality of *ESR1* mutations.

**Figure 3A** compares PFS between patients with and without b*ESR1* mutations. There were no significant differences in the PFS between patients with b*ESR1* mutation and those without (HR 0.59, 95% CI 0.20–1.71; log-rank p = 0.323; **Figure 3A**). **Figure 3B** illustrates PFS according to early (<6 months) versus late detection of b*ESR1* mutations. A total of three (18.8%) patients with b*ESR1* mutation were detected within 6 months after first-line ETx, representing a small subset of patients showing early emergence of b*ESR1* mutation. They all had distant recurrence with bone-only metastasis; two were treated with CDK4/6i + letrozole, and one was treated with fulvestrant as a palliative first-line ETx. Patients with b*ESR1* mutation within 6 months had a shorter PFS compared to those with b*ESR1* mutation detected after 6 months, although not statistically significant (HR 3.46, 95% CI 0.66–18.28; log-rank p = 0.120; median PFS, 5.5 vs. 53.6 months; **Figure 3B**). **Figure 3C** demonstrates the impact of mutation clearance versus persistent positivity on PFS. Patients with clearance of the b*ESR1* mutation in subsequent cfDNA analyses (81.3%, 13/16) were shown to have a longer PFS than in those with continuous detection (HR 0.87, 95% CI 0.17–4.35; log-rank p = 0.863; median, PFS 53.6 vs. 42.4 months; **Figure 3C**). **Figure 3D** compares PFS according to the presence or absence of polyclonal b*ESR1* mutations. Polyclonality of the b*ESR1* mutation was not significantly associated with PFS (HR 2.02, 95% CI 0.39–10.41; log-rank p = 0.393; **Figure 3D**). In exploratory multivariable Cox models including b*ESR1* status and clinical covariates (visceral metastasis or CDK4/6i use), no additional significant associations with PFS were observed (data not shown).

**Supplementary Figure S2** presents PFS among patients who experienced clinical progression, stratified by b*ESR1* mutation status. Among the 14 patients with clinical progression, eight had b*ESR1* mutations. There were no significant differences in PFS between *ESR1*-mutated and *ESR1*-wild-type patients (HR 0.66, 95% CI 0.21–2.01; log-rank p = 0.457; **Supplementary Figure S2**). The median PFS was 15.1 months (range, 10.2–34.8 months) for patients with b*ESR1* mutation and 9.3 months (range, 2.9–28.9 months) for those without the mutation. Among the patients with disease progression, four died.

**Figure 4:**
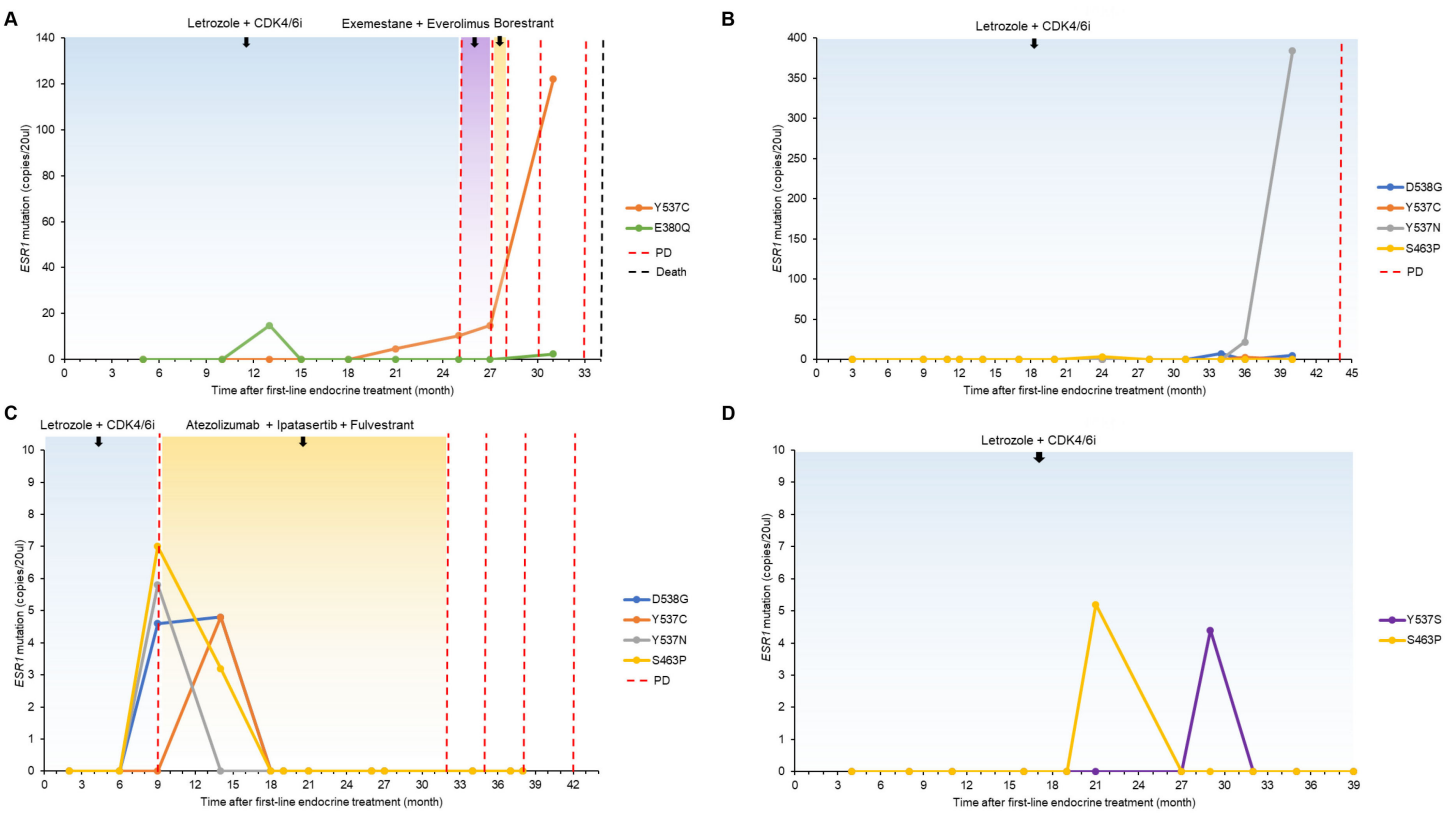
ctDNA trajectories in individual patients: **(A)** Patient DNAM033, **(B)** Patient DNAM043, **(C)** Patient DNAM014, **(D)** Patient DNAM027.

Representative individual mutation trajectories and corresponding clinical courses are shown in **Figure 4**. In patient DNAM033, b*ESR1* E380Q mutation was detected and eliminated; however, the disease progressed after the Y537C mutant copies gradually increased (**Figure 4A**). In patient DNAM043, the number of Y537N *ESR1* mutant copies increased before clinical disease progression (**Figure 4B**). Patient DNAM014 had multiple *ESR1* mutations detected at the time of disease progression, all of which cleared after switching to second-line treatment of atezolizumab + ipatasertib + fulvestrant, during which the disease remained stable for 23 months (**Figure 4C**). Patient DNAM027 showed transient detection of S463P and Y537S mutations, which became undetectable thereafter; the patient remained in a stable disease state (**Figure 4D**).

## 4 Discussion

In this longitudinal ctDNA analysis of patients with metastatic hormone receptor -positive/HER2-negative BC during first-line ETx, we reported the prevalence and trajectory of *ESR1* mutations and analyzed their correlation with clinical course and PFS. *ESR1* mutations were detected in 64.0% of plasma samples. Half (8/16) of the patients with b*ESR1* mutations showed clinical progression, among whom 4 were detected b*ESR1* prior to clinical progression. While the other half (8/16) of b*ESR1* positive patients displayed stable disease, all with subsequent plasma sample analyzed had b*ESR1* converted negative in the subsequent plasma sample.

We tested 268 samples from 25 metastatic BCs to investigate the serial changes in b*ESR1* mutations during palliative ETx and were able to illustrate some of the representative cases. DNAM033 and DNAM043 exhibited disease progression caused by b*ESR1* mutation which was detected prior to clinical disease progression with lead time of 4, 7 months respectively (**Figure 4A, B**). These two patients responded to first-line letrozole + CDK4/6i for > 24 months, yet acquired resistance developed by b*ESR1* mutation Y537C and Y537N respectively. Specifically, DNAM033 displayed gradual increase in number of b*ESR1* mutant copies before detection of clinical progression. These patients are the potential candidates who may benefit from ctDNA-guided early intervention, to fulvestrant or to new oral SERD agents.

DNAM014 also exhibited b*ESR1* acquired mutation under first-line letrozole + CDK4/6i. Polyclonal b*ESR1* mutation was detected at time of clinical progression and was converted negative as disease responded to subsequent treatment of fulvestrant + AKT inhibitor + PD-L1 inhibitor (**Figure 4C**). As disease progressed on the combined treatment, b*ESR1* stayed negative, implying the underlying mechanism to be other than *ESR1* hotspot mutation. If no known hotspot mutations are identified at this stage, it may be necessary to detect other newly acquired mutations using different panel testings. Since b*ESR1* mutations were confirmed early in these patients, or at the latest at the time of clinical progression, monitoring responses with serial blood tests could be advantageous, particularly when the mutations targeted during ETx for these tumors are clearly identified.

Finally, DNAM027 represents the subset of patients with b*ESR1* positive, yet responding to treatment till follow up (**Figure 4D**). Some even displayed multiple mutations, yet all patients with stable disease showed negative b*ESR1* conversion on the subsequent sample. As *ESR1*-mutant clones exist as in such a minor subclones, even it may mechanistically cause resistant to estrogen blockade strategy, it may not be sufficient to soley serve as a major driver population leading to elimination before becoming an actual ‘population’. In the control arm of PADA-1 study, substantial number of patients had a rising b*ESR1* mutation and remained progression-free under the same AI treatment without early intervention (17). While their serial *ESR1* data has not been released yet, it is thought that such a population will probably show a similar pattern as seen in our study. Previous studies also reported that *ESR1* mutation status changes during treatment (20, 21). It is currently unclear of the actual cutoff, number of negative tests or required intervals necessary to confirm negative and/or positive for clinical application during ETx. Interpretation should be made as a comprehensive course for each patient through serial follow-ups rather than by detecting ctDNA at a specific time point.

The prevalence of *ESR1* mutations was 64.0% in this study, which was higher than that reported in previous studies with various patient and treatment profiles (5, 7–9, 17). This may be because our study included many patients who received AIs (88.0%) for metastasis. The b*ESR1*-positive rate for patients on AI was 65.2% while 33.3% for the others. Schiavon et al. reported that *ESR1* mutations were present at a higher rate in patients treated with AIs in a metastatic setting and rarely in patients treated with AIs in an adjuvant setting (7). Additionally, many patients with bone metastases were included in this study. Fifteen (60.0%) patients had bone metastases, 11 patients had bone-only metastases, and 4 had bone metastases with other metastatic sites. Fribbens et al. reported that *ESR1* mutations are significantly associated with bone metastasis (9). *ESR1* mutations are known to emerge mainly during secondary endocrine resistance. This pattern was consistent with our cohort: none of the patients with primary endocrine resistance harbored b*ESR1* mutations, whereas 70% of those with secondary resistance did. This distinction further supports the notion that *ESR1* mutations develop under the selective therapeutic pressure.

Early and sustained detection of mutations in ctDNA tended to result in shorter PFS, although this was not statistically significant. Given the limited sample size of our cohort, the lack of statistical significance may reflect insufficient power rather than true biological similarity between groups. In this study, only three patients exhibited b*ESR1* detection within 6 months, representing a very small subset. Although these patients appeared to have shorter PFS, the limited sample size precludes any firm conclusion, and these findings should be interpreted with caution. Nevertheless, together with emerging evidence from SERENA-6 (13), our observations support the potential value of early intervention strategies following b*ESR1* detection. To illustrate how serial ctDNA monitoring may guide treatment decisions in practice, we added a conceptual framework summarizing potential clinical decision points based on b*ESR1* dynamics (**Supplementary Figure S3**). This framework is exploratory and intended to contextualize our observations within emerging ctDNA-guided treatment strategies. Future prospective studies are needed to validate the timing thresholds, switching criteria, and clinical utility of ctDNA-informed treatment adaptation. Polyclonality of the b*ESR1* mutation was not associated with PFS, which is consistent with previous studies (9, 21). While *ESR1* mutations are considered to be associated with poor PFS, b*ESR1* positivity was not prognostic within our study population (22, 23). This finding may be explained by the small number and the heterogeneity nature of the patients, especially regarding exposure to AI. Also, our analysis included patients with b*ESR1* mutation positive at any time, regardless of negative conversion, while most studies evaluated the baseline b*ESR1* status which makes it challenging to compare the prognostic impact. However, we were indeed able to observe that b*ESR1* mutations arise as a result of acquired resistance from AI treatment, ultimately leading to the manifestation of clinical progression which implies the necessity of b*ESR1* monitoring especially during AI treatment. In the PADA-1 trial, there was a PFS benefit for the early switch of ETx before clinical relapses through serial ctDNA monitoring of rising b*ESR1* mutations (17). Moreover, recent trials investigating novel oral SERDs have also demonstrated significant improvements in PFS among patients with *ESR1* mutations (11–13). These findings support the clinical utility of *ESR1* mutation detection and treatment adaptation based on ctDNA monitoring.

We compared ctDNA-guided molecular progression with radiographic clinical progression. Previous studies have shown the possibility of the early detection of ctDNA before disease progression (24, 25). The predicted lead time of hormone receptor-positive, HER2-negative BCs from previous studies are 10 months (26, 27). In our study, 50% of the 16 b*ESR1* positive patients showed subsequent/simultaneous clinical progression, and the other 8 patients displayed long term response to ETx. There were 4 cases where b*ESR1* was detected simultaneously with clinical progression, and 4 cases where b*ESR1* mutation was detected prior to clinical progression. As most patients had blood sampling taken at times of every radiologic evaluation with some in between radiology, we were able to directly assess the actual lead time of ctDNA guided molecular progression to radiologically detected clinical progression. The simultaneous clinical progression rate at the time of b*ESR1* positivity in our study was 50% (4/8), which was higher than the 21.5% (60/279) of simultaneous clinical progression reported in the PADA-1 trial (17). The mean lead time was 7.6 months in our study and this can be translated into primary PFS of 5.8 months in control arm of PADA-1 trial. These differences can be explained by several distinctions between the two studies. In the PADA-1 clinical trial, blood sampling intervals were strictly set at two months, whereas radiographic evaluation intervals were more flexible, allowing investigators discretion to perform them within four cycles, while we have a matched set of 2-3 monthly blood and radiology evaluation. Overall, given that ctDNA-guided early intervention prolonged PFS in the PADA-1 clinical trial, is anticipated that shortening the interval between ctDNA testing may be more beneficial. Furthermore, this finding also suggests that the ctDNA guided monitoring may be a potential substitute or supplementary for routine radiological response monitoring.

*ESR1* mutation is an acquired mutation after ETx as a result of therapeutic pressure. Most clinical trials on ETx enrolled patients who received first-line treatment for at least six months; therefore, *ESR1* mutation testing was also performed six months after first-line ETx (28, 29). However, our study examined blood samples collected within 6 months of starting palliative ETx and revealed that patients who developed b*ESR1* mutations within 6 months of palliative ETx tended to have a shorter PFS (n = 3). Although the number of affected patients was small, it is important to initiate ctDNA monitoring as patients who detect positive for b*ESR1* within 6months displayed worse outcome.

In this study, among the 14 patients with disease progression, 8 patients with b*ESR1* mutations and six patients progressed without b*ESR1* mutations (**Figure 2**). Although a significant proportion of cases with endocrine resistance are associated with *ESR1* mutations, other resistance mechanisms that are mutually exclusive to *ESR1* have also been identified (30–32). It can be assumed that other resistance mechanisms may have contributed to the disease progression in these patients. Our findings further highlight the heterogeneity of endocrine resistance. Some patients harboring b*ESR1* mutations maintained prolonged clinical stability, whereas others without detectable b*ESR1* mutation nevertheless experienced disease progression. This pattern illustrates that *ESR1* mutations alone cannot fully account for endocrine resistance and should be interpreted within the broader context of coexisting resistance pathways. As *ESR1* exists as subclone in most of cases, there could be quite a lead time between radiologic progression which in fact, is one of the reasons how b*ESR1* provides long window of opportunity period for early switching. Moreover, previous study has shown that *ESR1* mutation subtypes show differential ligand-independent activity and variable responsiveness to ER-targeting agents (10), which may help explain the variability in clinical response observed in our study. And importantly, as *ESR1* mutation accounts for ~20% of endocrine resistance, patients may exhibit progression without b*ESR1* mutation and should be assessed with other targetable alterations, such as PI3K/AKT pathway alteration (30). Prognosis of the patients with disease progression according to the b*ESR1* positivity exhibited numerically longer PFS for b*ESR1* positive patients than those without the mutation (15.1 months vs. 9.3 months, p = 0.457; **Supplementary Figure S2**). This is consistent with previous findings that there is concrete subset of populations regardless of *ESR1* mutation who display dreadful outcome of PFS <3months for both standard and novel agents therapy (11, 33). Currently, genomic testing guided targeted agents provide an opportunity for precision medicine, and guidelines recommend mutational profiling prior to deciding therapeutic decision making (14, 34). Yet, these tumor-agnostic panel testing have limited role in response monitoring during treatment.

This study had several limitations. First, this study involves a small number of patients, limiting its statistical power of relevance. Therefore, the non-significant results observed should be interpreted as potentially underpowered findings rather than the absence of a true effect. Nevertheless, we found a trend toward worse PFS in patients with early detection of b*ESR1*, sustained b*ESR1*. These findings highlight the need for identifying these mutations during ETx. In addition, only 25 of the 78 eligible patients met the requirement for serial sampling every 2–6 months, introducing potential selection bias toward patients with consistent clinical follow-up. This reflects the real-world challenges of implementing serial ctDNA testing and highlights the need for feasibility evaluation in future prospective studies. Second, although proportional hazards assumptions were visually checked and no major violations were observed, the limited sample size constrains confidence in these assessments. Similarly, while Cox regression was performed, the number of progression events restricted the ability to adjust for additional covariates without risking model overfitting; therefore, residual confounding cannot be excluded. Third, this study was a retrospective analysis of prospectively collected samples from consecutively enrolled patients and was not randomized. Therefore, it is a heterogeneous population and there may have been a selection bias. For example, our study includes patients who weren’t given the current standard of choice regimen for various reasons eg. enrolled prior CDK4/6i era. However, in this study, we aimed to show the clinical results in a real-world setting. Finally, only *ESR1* mutations were identified. However, as other resistance mechanisms may have contributed to disease progression, there are limitations in interpreting the clinical course of each patient based solely on these results.

Our study has strength of sequentially following each patient with samples from multiple time points throughout palliative treatment course. Previous studies that examined b*ESR1* mutations in metastatic BC showed baseline only or after 1-2 times after short duration of treatment (20, 35). Though the number of patients is small, we performed an in-depth analysis of the long-term clinical course and mutational status within, demonstrating the development of resistance and disease progression during ETx in clinical practice. Also, we used 2ml of processed plasma from 4ml of whole blood draw from EDTA tubes. Many ctDNA assays require 20ml of whole blood to overcome limit of detection issue but 20ml blood draw on top of other samples eg. complete blood count for actual treatment may be quite burdensome for patients especially when done every timepoints for monitoring. Strek tubes enable stable preservation of the cfDNA, yet, developing a system for blood processing could be more efficient in the long term, considering the societal healthcare costs. We have specifically chosen seven *ESR1* hotspot mutations for this study for this population undergoing palliative ETx. In order to address the benefit of early intervention depending on the result of ctDNA, we are waiting for the overall survival data of the PADA-1 trial, along with other trials testing new oral agents. Deciding the optimal interval of testing and standardization of assay, plasma preparation would be critical for clinical application. For patients with b*ESR1* negative at time of clinical progression, targeted sequencing of both/either metastatic tumor and/or ctDNA may be beneficial to identify the mechanism causing the actual resistance and disease progression, in order to decide subsequent lines of therapy.

## 5 Conclusion

By applying a sensitive method ddPCR, we were able to detect *ESR1* hotspot mutations from serially collected plasma samples from a subset of patients with hormone receptor -positive/HER2-negative metastatic BC during palliative ETx. Our results suggest that early detection of b*ESR1* within 6 months of palliative ETx and sustained b*ESR1* were associated with shorter PFS. Among all b*ESR1* positive patients who displayed clinical progression, 4 were detected prior to clinical progression, which may be a window of opportunity for early intervention. ctDNA-guided monitoring may have a role as a substitute or a supplementary of radiologic response monitoring during palliative ETx.

## Data Availability

The original contributions presented in the study are included in the article/**Supplementary Material**. Further inquiries can be directed to the corresponding author.
