# Supplementary material for: Serial analysis of ESR1 mutations in cell-free DNA from hormone receptor-positive, HER2-negative metastatic breast cancer during palliative endocrine therapy
Source: Front Oncol. 2026 Jan 6;15:1709317. doi: 10.3389/fonc.2025.1709317 (PMC12815856; doi:10.3389/fonc.2025.1709317)
Supplement: Supplementary file 1 [file DataSheet1.pdf]

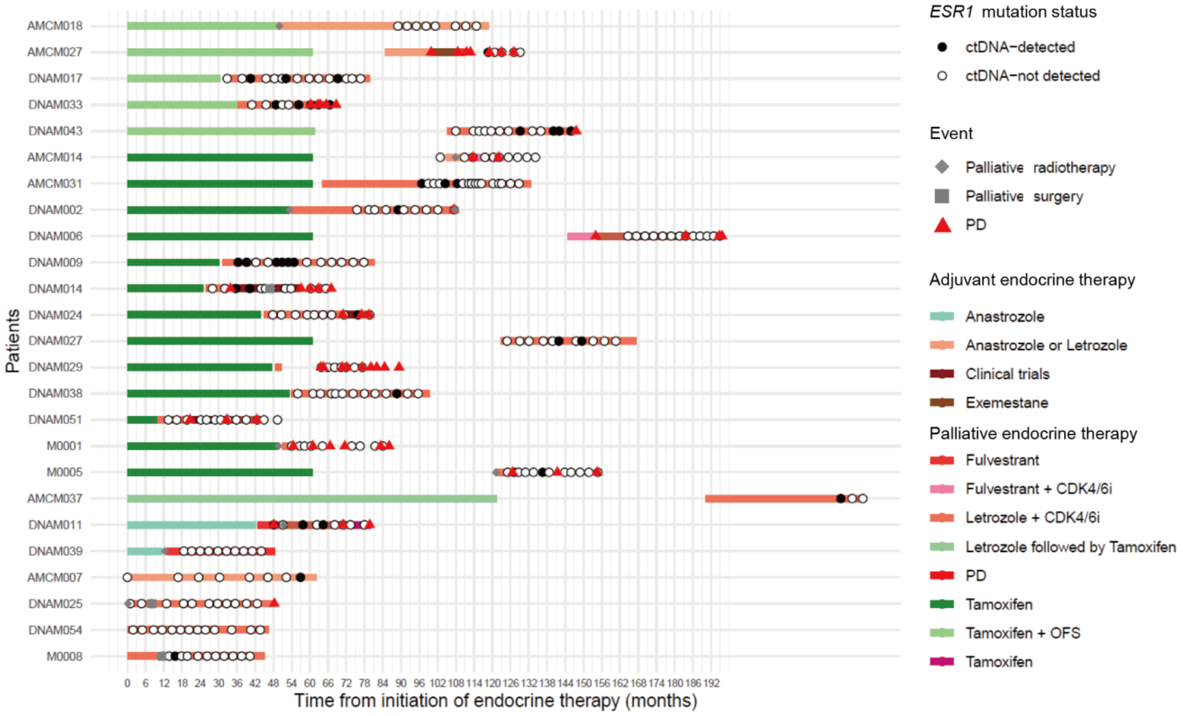

Supplementary Figure S1. Swimmer plot showing the *ESR1* mutation status and clinical information of individual patients after initiation of endocrine therapy.

PD progressive disease

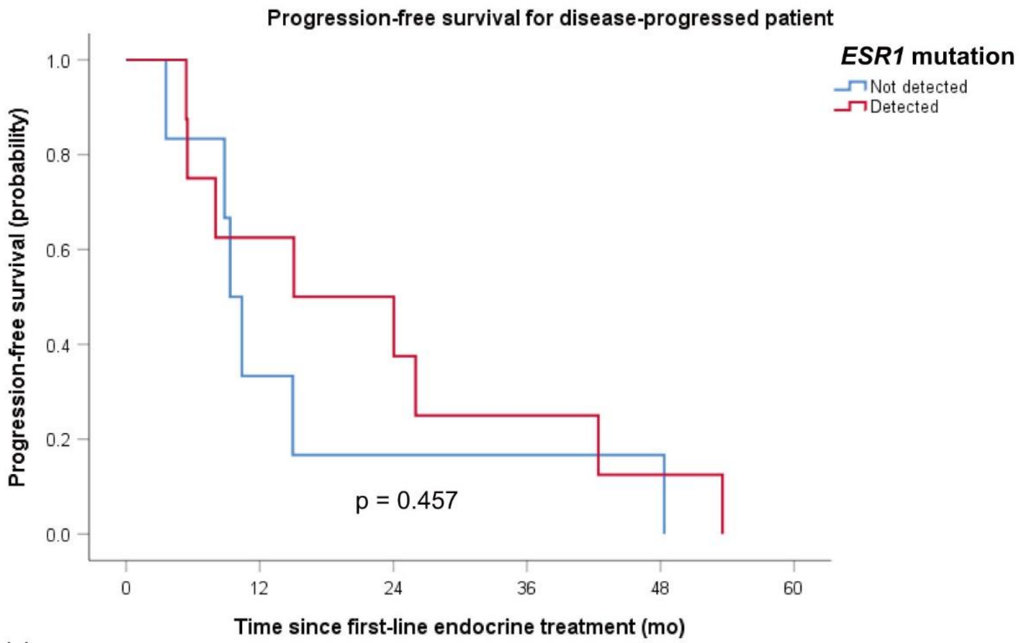

No. at risk

Not detected	6	2	1	1	1	0
Detected	8	5	4	2	1	0

Supplementary Figure S2. Kaplan–Meier curves of progression-free survival for disease-progressed patients according to *ESR1* status.

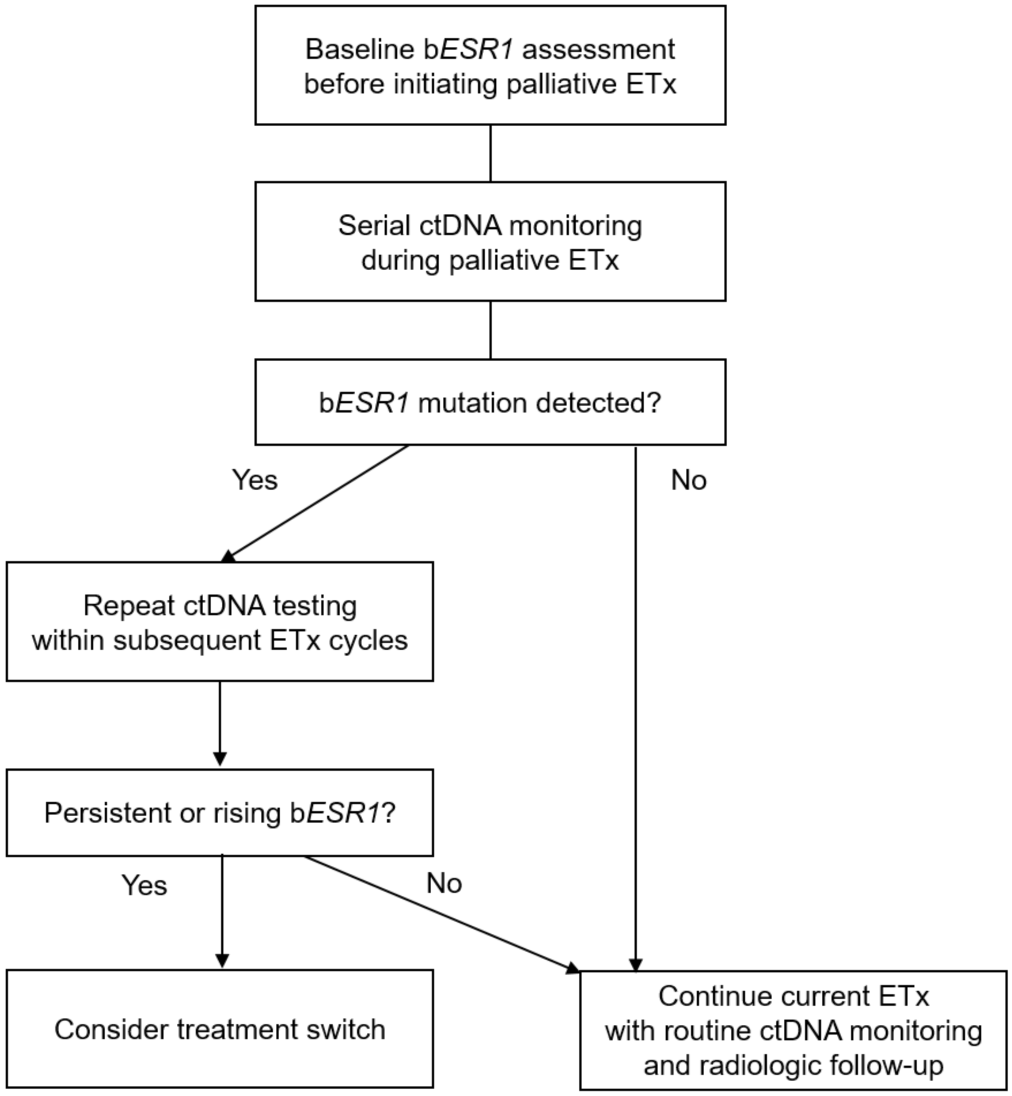

Supplementary Figure S3. Conceptual decision framework for potential ctDNA-guided endocrine treatment adaptation based on serial b*ESR1* monitoring.

b*ESR1* blood *ESR1* mutation, ETx endocrine therapy
