# Supplementary material for: Serial analysis of ESR1 mutations in cell-free DNA from hormone receptor-positive, HER2-negative metastatic breast cancer during palliative endocrine therapy
Source: Front Oncol. 2026 Jan 6;15:1709317. doi: 10.3389/fonc.2025.1709317 (PMC12815856; doi:10.3389/fonc.2025.1709317)
Supplement: Supplementary file 2 [file Table1.docx]

|  | *ESR1* mutation | No. of patients with mutation | No. of samples with mutation | No. of patients with *ESR1* polyclonality | No. of samples with co-existence of multiple *ESR1* mutations | Range of detected mutation (copies/20ul) | Metastatic site | | Prior adjuvant endocrine therapy regimen | | Clinical outcome with radiologic assessment | | |
| --- | --- | --- | --- | --- | --- | --- | --- | --- | --- | --- | --- | --- | --- |
|  |  |  |  |  |  |  | Bone-only metastasis | Visceral metastasis | Aromatase inhibitor | Tamoxifen | No. of patients with b*ESR1* mutation and subsequent  /concurrent clinical progression (%) | No. of patients with b*ESR1* mutation detected and spontaneous clearance (%) | No. of patients with clinical progression without b*ESR1* mutation (%) |
| *bESR1* mutation detected at any time  (n = 16) | D538G | 10 | 17 | 8 (80%) | 15 (88.2%) | 2.4–7.4 | 3 | 5 | 1 (12.5%) | 7 (87.5%) | 3 (33.3%) | 6 (66.7%) | N/A |
|  | S463P | 8 | 14 | 8 (100%) | 14 (100%) | 2.2–9.6 | 2 | 6 | 1 (12.5%) | 7 (87.5%) | 1 (12.5%) | 7 (87.5%) |  |
|  | Y537C | 8 | 12 | 7 (87.5%) | 11 (91.7%) | 2.2–122 | 2 | 6 | 1 (12.5%) | 7 (87.5%) | 1 (12.5%) | 7 (87.5%) |  |
|  | Y537N | 5 | 6 | 4 (80%) | 5 (83.3%) | 3.4–384 | 2 | 2 | 2 (40%) | 3 (60%) | 1 (20%) | 4 (80%) |  |
|  | Y537S | 2 | 2 | 1 (50%) | 1 (50%) | 3.4–4.4 | 0 | 2 | 0 (0%) | 2 (100%) | 1 (50%) | 1 (50%) |  |
|  | E380Q | 1 | 2 | 1 (100%) | 2 (100%) | 2.4–14.8 | 0 | 1 | 0 (0%) | 1 (100%) | 1 (100%) | 0 (0%) |  |
| *bESR1* mutation never detected at any time  (n = 9) | | N/A | N/A | N/A | N/A | N/A | 6 | 2 | 2 (28.6%) | 5 (71.4%) | N/A | N/A | 6  (66.7%) |

**Supplementary Table S1.** Summary of b*ESR1* mutation subtype characteristics and related clinical trajectories.
